# A locally initiated and executed measles outbreak response immunization campaign in the nylon health district, Douala Cameroon 2011

**DOI:** 10.1186/1756-0500-6-100

**Published:** 2013-03-16

**Authors:** Gerald Etapelong Sume, André Arsène Bita Fouda, Marie Kobela, Salomé Nguelé, Irène Emah, Peter Atem

**Affiliations:** 1 Regional Delegation of Public Health for the Littoral, PO Box 106, Douala, Cameroon; 2 The Central Technical Group for the Expanded Programme on Immunization, PO Box 2084, Yaoundé, Cameroon; 3 Nylon Health District Service, PO Box 5458, Douala, Cameroon

**Keywords:** Measles, Outbreak, Response immunization campaign, Nylon health district

## Abstract

The Cameroon health system is divided into central, intermediate and peripheral levels. Of the 43 health districts with a measles outbreak in Cameroon in 2011, only the Nylon Health District organized a documented outbreak response immunization. We present the methods and results of the response campaign solely shouldered by the district and intermediate level. The risk group, targets and neighborhoods to be vaccinated were identified after a detailed analysis of initial cases. The intermediate level defined strategies, provided logistics, capacity building and 41% of the operational budget while 59% was completed by the peripheral level. Microsoft Office Excel 2007 was used to estimate coverage rates and to draw an epidemic curve. The response immunization campaign was organized on the 14^th^ epidemiological week, 10 weeks after the onset of the outbreak which ended 11 weeks thereafter. A total of 15867(108.5%) children aged 9-59 months were vaccinated in five health areas at a direct cost (vaccines excluded) of 71.34FCFA ($0.143) per vaccinated child. An additional 824 children aged 9-59 months were vaccinated around the residence of notified cases in neighborhoods which were not involved in the response campaign. The number of cases after the response campaign was lower than before. Once vaccines are available, prompt outbreak response campaigns can be organized at operational level to obtain commendable results instead of depending solely on international organizations or central levels. Decision makers at the intermediate and operational levels should redeploy available funds during emergencies to prevent the development of extreme public health conditions.

## Discussion

Nylon Health District, one of the six districts of the Littoral region with a measles outbreak in 2011 took the challenge to personally organize an outbreak response campaign. As soon as the intermediate level was informed by the central level on the advent of a measles outbreak in February 2011 [three laboratory confirmed Measles IgM positive cases from the Nylon Health District in the month of February 2011, which is within 4 weeks], they in turn informed the district team. A series of events followed the confirmation of the outbreak leading to a response immunization campaign immediately followed by specific interventions around notified cases in health areas not involved in the response immunization campaign.

This paper presents the procedure the staff of the Nylon Health District and the intermediate level used to organize and auto-finance a measles outbreak immunization response campaign as well as the results of the campaign and compares the trend of cases reported by all nine health areas of the district to those reported by the health areas that benefitted from the said campaign using an epidemic curve. The purpose for which is to appeal to decision makers at intermediate and operational levels to mobilize local resources and organize timely outbreak response immunization campaigns in certain situations so as to minimize morbidity, mortality and duration of vaccine preventable diseases outbreaks instead of waiting indefinitely for financial assistance from international organizations and the central level. Documenting our methods and results in a scientific journal constitute best practices that should be used by other programme managers for posterity.

We found that if vaccines and consumables are available, it is possible to locally and promptly plan, finance, implement and evaluate an effective mass measles outbreak response immunization campaign by using available local resources so as to reduce morbidity and mortality. This illustrates a good example of a well coordinated rapidly organized measles outbreak immunization response campaign solely sponsored by the operational and intermediate levels after early detection and confirmation of a measles outbreak, with keen situational assessment as suggested by the new World Health Organization (WHO) recommendations for the use of vaccines to manage measles outbreak in countries with measles mortality reduction [1].

The present structure of the Expanded Programme on Immunization (EPI) in Cameroon follows the organization of the health system with a central technical group found in the central level, a regional body found at the intermediate level, health areas and districts found at the peripheral level. Policies are defined at the central level and implemented at the peripheral level. There was a countrywide measles outbreak in 2009 that led to the organization of a nationwide mass measles campaign targeting children aged 9 to 59 months. In 2010, ten measles outbreak (ten health districts) were reported in the northern part of the country with over eight hundred cases and a case fatality of 2.2% [2]. In 2011, unpublished data from the Central Technical group of the EPI shows that, about 43(24.9%) of the 173 health districts in the country had a measles outbreak with 502 laboratory confirmed measles cases, but unfortunately no mass response immunization campaign was organized. This is because the main sponsoring organizations had planned for a campaign in 2012. Thus all the outbreaks were allowed to follow their natural course until May 2012 when the nationwide outbreak response immunization campaign was organized.

This is an unprecedented success story in Cameroon as never an intermediate and/or operational level had solely organize, finance and document a mass immunization response campaign to an outbreak due to a vaccine preventable disease. Mass immunization response campaigns in Cameroon have always been initiated by the central level and totally or partially financed using direct funds from international organizations and/or the state. Due to the absence of funds from these organizations in 2011, no nationwide mass measles immunization response campaign was organized despite the fact that 43 health districts reported a measles outbreak. The response was delayed till May 2012 when the funds from international organizations were available. According to unpublished data from the Central Technical Group of the Expanded Programme on Immunization, there were 69 districts with a measles outbreak by the onset of the immunization response campaign on the 2^nd^ of May 2012. Probably this would have been lesser if an outbreak immunization response was organized in 2011.

Previous knowledge and the position of WHO has shifted from principally advocating for adequate management of cases during measles outbreak [3,4] to outbreak immunization response campaign in countries with measles mortality reduction goal like Cameroon [1,5]. Measles outbreak response immunization campaign has been shown to reduce measles morbidity and mortality during confined as well as outdoor outbreaks [6,7]. The effectiveness of such campaigns depend on the timeliness of the response. It has been demonstrated both in real life and in simulation models that the shorter the time interval between the index case and the response campaign, the more effective is the campaign in terms of reduction in the size (morbidity and mortality) of the outbreak [8-10].

Although the outbreak immunization response campaign was organized when the number of cases was on a decrease, we can’t say with certainty if there wasn’t going to be another series of smaller outbreaks. The campaign still played a major role to reduce the morbidity of the outbreak as just 20% of cases were reported after the campaign in health areas involved in the said campaign and just 09 cases reported more than two weeks after the campaign in health areas not involved in the mass campaign. The reduction in the spread of the infection to health areas not involved in the mass campaign may also be due to the specific outreach vaccination strategy around cases. This strategy was put in place bearing in mind the notion that vaccination with the measles vaccine within 72 hours post exposure to the virus is known to prevent disease or reduce disease severity [11].

Apart from funds (hospital budget) given to public health facilities twice a year by the state, hospitals also make money from their day to day activities. Their income is divided into “Revenue Kept Aside” and “Cost Recovery”. Revenue Kept Aside is money they keep for twelve months and use at the beginning of the following year after the elaboration of a budgeted plan whereas Cost Recovery Funds are used for the hospital monthly up keep. Therefore the Chief Medical Officers were willing to take money from hospital funds on the instructions of the intermediate level to make the response possible. It is easier to make government institutions contribute for such a mass action because the administration has direct authority over them compared to private for-profit or private not-for-profit institutions.

The average cost of vaccinating a child was less than a 100FCFA ($0.2) excluding the cost of vaccines and consumables. It is worth noting that, vaccines and consumables used for the campaign in the Nylon Health District were taken from the trimester stock available at the regional store under the instructions of the central level, hence not included in our estimate. Vaccines and consumables would constitute the bulk of the cost of a mass outbreak immunization response campaign like ours. Thus once vaccines and consumables are available, the running cost of a response immunization campaign can be taken care of at operational and intermediate levels. Once this is understood by the personnel at the operational level, timely immunization response campaigns to outbreaks due to vaccine preventable diseases becomes feasible to reduce their morbidity, mortality and duration.

Interventions carried out around reported cases in the four health areas which were not involved in the immunization response campaign were used as a strategy to control the rapid spread of the outbreak in those neighborhoods. This permitted us to vaccinate almost a thousand more infants aged 9 to 59 months against measles and to help limit the spread of the outbreak.

The outbreak was confirmed on the 4^th^ epidemiological week of 2011 and the outbreak immunization response campaign started on the 14^th^ week. This implies 10 weeks after onset. The 10 weeks may be greater than the 7 weeks time interval for maximal reduction in the size of an outbreak in a simulation model, but it is also lower than the 13 weeks no difference time limit in the same study described by Bonacic and colleagues [8]. Considering the African context were measles outbreaks can be very long and persistent and evolving in small new outbreaks over time [6,9,12] 10 weeks can still be a good enough time to organize an effective outbreak immunization response. More so, if the operational level is encouraged to organize response campaigns with local funds, it is possible to further reduce this interval. In the case of the Nylon Health District, we could have gained about 4 weeks if we subtracted the time needed to wait for feedback of confirmed measles cases from the central level and the uncertainty surrounding the availability of funds and possible organization of a mass immunization response campaign sponsored by international organizations and the state. This puts us below the 7 weeks and 60 days time interval between onset of a measles outbreak and immunization response campaign to obtain maximum reduction in morbidity and mortality as described by Bocicnic and Grais respectively [8,10]. Approximately 10 weeks was the time interval needed to organize the mass outbreak measles immunization response campaign in the Far North region of Cameroon in 2009 during the long lasting measles outbreak with the partner support [6].

The overall vaccination coverage of the response campaign was over 100%. This is because the denominator was an estimate from previous campaigns in various neighborhoods involved in the response campaign. Only the total population of Bonadiwoto was involved in the campaign. More so we could not also refuse children aged 9 to 59 months that presented at vaccination sites to benefit from the vaccines even if they lived in neighborhoods not selected for the campaign. On the other hand, the low vaccination coverage in children aged 9-11 months is not only due to reluctance of mothers taking these young kids to vaccination sites but both areas had few teams to cover the affected area. More so the Oyack neighborhood is very inaccessible with hills, valleys and road paths making movement difficult for both teams and beneficiaries. Up to 204(1.4%) of the vaccinated infants had never received a measles containing vaccine before the campaign, 107(52.5%) of whom were above 12 months old. Everything being equal, according to our EPI programme directed towards children aged 0-11 months, these children were supposed to have received measles vaccine which is administered at 9 months of age. This shows that despite the high measles vaccine coverage in the Nylon Health District (96% for 2010, unpublished data), there are still unvaccinated children in the slums of the district.

### Limitations

The immunization response campaign did not cover the whole district. Children aged 6 to 14 years with an attack rate of 2/10000 inhabitants were not included in the response campaign. Nonetheless the neighborhood and age group chosen for the response campaign was done from a detailed assessment and resources were limited. Specific interventions around cases in health areas not involved in the campaign were also used to address the above mentioned limitation. Except for the Bonadiwoto health area, the target populations of the other health areas were estimates got from previous campaigns. These estimates were not exact and partly explained why some of the coverages were above 100%.

### Pre response phase

#### Setting

Created in 1992, the Nylon Health District is one of the six health districts in the cosmopolitan city of Douala (the economic capital of Cameroon) and the 2^nd^ most populated in the Littoral region. It is an urban slum located in the south eastern part of the city with an estimated population of 444010 inhabitants in 2011, distributed over 700 hectares of land. It is divided into nine health areas and had 52 vaccinating health facilities (public, private for-profit, private not-for-profit) in 2011.

#### Initial analysis

As soon as the intermediate level was informed about the outbreak, initial active search was done in the health facilities and neighborhoods of the district from the 8^th^ to the 13^th^ epidemiological week. This came out with 48 suspected cases. Of these, 30(63%) were females and 28(58.3%) aged 9 to 59 months. The Bonadiwoto health area alone had 30(63%) of the cases. Of the 48 cases, 26(54.2%) declared to have been vaccinated against measles but only 4(8.3%) children could have their vaccination status confirmed with a vaccination card or hospital booklet.

#### Choice of response sites

The sites for the response campaign were chosen with respect to the age specific attack rates and the geographic distribution of the first 48 cases. In all, the global district attack rates were 4/10000, 5/10000 and 2/10000 inhabitants for the less than 9 months, 9 to 59 months and more than 59 months age groups respectively. From the initial analysis done on the 29^th^ of March 2011, of the eight health areas who reported at least a case, three (Diboum, Ndogpassi Zone de Recassement and Ndogpassi Center) did not have cases in the 9 to 59 months age group. The attack rates in infants aged 9 to 59 months in decreasing order in the remaining five health areas were: 36/10000 inhabitants (Bonadiwoto), 7/10000 inhabitants (Barcelone), 4/10000 inhabitants (Oyack), 3/10000 inhabitants (Boko) and 2/10000 inhabitants (Soboum). These five health areas were chosen for the response campaign in favour of the most affected age group (9 to 59 months) in terms of proportion and attack rate.

#### Estimation of targets for the response campaign

According to the 2005 national census of Cameroon, infants aged 9 to 59 months represents 12% of the total population in the Littoral region [13]. Since 63% of the cases came from Bonadiwoto health area, the entire health area was involved in the response immunization campaign. The target zone for the remaining four health areas were determined with the district team around the suspected cases and the population size estimated from previous mass campaigns results per neighborhood. The estimated target population for the response campaign for the five health areas was 14627 infants aged 9 to 59 months.

#### Capacity building and strategies enforced

A training session involving health personnel from all health facilities within the district was organized by the intermediate level in collaboration with the district service to present the strategies put in place by the region to arrest the outbreak. The region accompanied by the district team developed and implemented the following strategies:

– Setting up a District Coordination Team with clearly elaborated terms of reference under the direction of the District Medical Officer (DMO);

– Reinforcing surveillance at all levels involving community and technical staff;

– Organizing and harmonizing symptomatic case management including vitamin A administration and patient registration;

– Immediate vaccination of contacts aged 9-59 months living in the same house and a circumference of two houses around a suspected case (an average of twenty houses) by two nurses identified from pilot health facilities. This started on the 6^th^ of April 2011 and concerned only the four health areas which were not involved in the immunization response campaign;

– Reinforcing Routine vaccination of all children aged 0 to 59 months found in health facilities or during outreach activities;

– Sensitization at all levels using mass media, social mobilization and health education in hospitals;

– Measles outbreak response immunization campaign directed towards infants aged 9 to 59 months in highly affected neighborhoods of five health areas. The campaign was done on the 4^th^ and 5^th^ of April 2011. Each team was made up of three persons (a vaccinator, a registrar and a social mobiliser). Social mobilization started on the 3^rd^ of April and lasted three days.

#### Elaboration of the budget and fund raising

We budgeted 44 vaccinators, 44 registrars and 44 social mobilizers at 2000FCFA ($4) per person per day for perdiem, five regional supervisors (10000FCFA/day or $20 perdiem per person), six district supervisors (5000FCFA/day or $10 perdiem per person), five drivers from the region (5000FCFA/day or $10 perdiem per person) and five drivers from the district (3000FCFA/day or $6 perdiem person). Also budgeted was transport fare for the teams and fuel for a total of 298000FCFA ($596). The total cost of the outbreak immunization response campaign was 1232000FCFA ($2464); of which 100000FCFA ($200) was meant for teams carrying out specific post campaign intervention around cases in areas not concerned by the response immunization.

The 1232000FCFA ($2464) was shared amongst and raised by public health facilities of the NHD. All the health facilities were instructed by the intermediate level to chip in their contribution from their “Cost Recovery Funds”. The intermediate level contributed 500000FCFA ($1000), the Nylon District Service 72000FCFA ($144), the Nylon District Hospital 300000FCFA ($600), the Sub-Divisional Medical Centres of Soboum, Bonadiwoto, Oyack and Diboum 165000FCFA ($330) each.

#### Logistics

The regional store provided 16100 doses of measles vaccine with an equivalent number of diluents and auto-disabled syringes for the response campaign. Six 500 g of cotton and 161 safety boxes were also provided by the regional store. A pick-up was sent from the intermediate level to each of the five health areas involved in the campaign. The district was asked to use vaccine from its stock in case of stock out and also to provide vaccines for specific interventions around cases. Vitamin A was provided by the stock in health facilities and was administered to cases in the hospitals as defined in the strategies. Vitamin A was not administered in the community during the mass campaign.

#### Data collection and analysis

During the training, a series of documents (hard copies) were given to each hospital represented at the meeting so as to ease and harmonize identification and investigation of cases, case management and reporting. These included a measles line list, measles notification form, measles outbreak investigation form, case definition and detailed elaborated strategies. Electronic copies of the same documents were given to the district service. A tally sheet was used to document the number of infants vaccinated during the campaign and during case specific response interventions. During the campaign, we had a daily evaluation and synthesis meeting at the district service. The specific response intervention data was later on transmitted by pilot health facilities to the district service whenever such an intervention was done.

Data was entered and analyzed using Microsoft Office Excel 2007. The line listing was typed in an Excel sheet and the epidemic curve of the entire outbreak as well as that for the five health areas with a response immunization campaign presented. The proportion of infants vaccinated during the response campaign was presented using a frequency table. The number of health facilities and participants who took part in the training was also reported. The direct cost per child vaccinated (excluding cost of buying vaccines and consumables) was calculated by dividing the amount of money used for the response campaign by the number of children vaccinated during the campaign.

### Response phase

The training took place on the 29^th^ of March 2011 (13^th^ epidemiological week) and the immunization response campaign on the 4^th^ and 5^th^ of April 2011 (14^th^ epidemiological week). Meanwhile specific vaccination around notified cases started on the 6^th^ of April 2011 (14^th^ epidemiological week) and ended on the 30^th^ of May 2011 (22^nd^ epidemiological week). Fifty one health personnel from 33 health facilities (8 public hospitals, 25 private for-profit and private not-for-profit) and nine dialogue structure members attended the training on the strategies put in place by the region to arrest the outbreak.

#### Measles outbreak immunization response campaign performance

As illustrated in Table 1, a total of 15867 children aged 9 to 59 months were vaccinated out of an estimated target of 14626 children given a coverage rate of 108.5%. The Bonadiwoto health area with the largest target, registered the lowest coverage rates in children aged 9 to 11 months 379(62.5%) and 12 to 59 months 4854(104.3%) respectively. Whereas Soboum 693(141.2%) and Boko 2151(129.7%) health areas had the highest coverage rates in the 9 to 11 months and 12 to 59 months age groups respectively. There were 204 zero doses of which 107(52.5%) were children aged 12 to 59 months. Most of the zero dose children were from the Soboum health area 106(52%) followed by Bonadiwoto health area 43(21.1%). The wastage rates for vaccines and consumables were within normal limits and 129 safety boxes were used and incinerated. The direct cost (cost of vaccines excluded) per child vaccinated was estimated at 71.34FCFA ($0.143).

**Table 1:** Measles outbreak immunization response campaign performance in 5 health areas in the NHD, Douala-Cameroon 2011

**Health Areas**	**Target population**			**Vaccinated against measles**									**Vaccine and consumables used**					**Wastage rates (%)**			
	**Age groups (months)**			**Number of vaccinated children**					**Vaccination coverage (%)**				**Doses**	**Diluent**	**Syringes**		**SB and Inc.**			**ADS**	**Dilution**
	**9 -11**	**12-59**	**9 - 59**	**9 -11 m**	**0 dose**	**12-59 m**	**0 dose**	**9 - 59 m**	**9 -11 m**	**12-59 m**	**0 dose**	**9 - 59 m**	**MV**	**MVD**	**ADS**	**Dilution**		**MV**	**MVD**		**Syringe**
Barcelone	273	1908	2181	147	10	2298	17	2472	57,5%	121,3%	1,2%	113,3%	2500	2500	2570	250	18	1,1%	1,1%	3,8%	1,1%
Boko	237	1663	1900	197	2	2151	6	2356	84,0%	129,7%	0,4%	124,0%	2360	2360	2373	236	22	0,2%	0,2%	0,7%	0,2%
Bonadiwoto	666	4660	5326	379	37	4854	6	5276	62,5%	104,3%	0,8%	99,1%	5360	5370	5543	536	50	1,6%	1,8%	4,8%	1,6%
Oyack	132	925	1057	50	5	1153	13	1221	41,7%	126,1%	1,7%	115,5%	1230	1230	1223	123	10	0,7%	0,7%	0,2%	0,7%
Soboum	520	3642	4162	693	41	3743	65	4542	141,2%	104,6%	2,5%	109,1%	4710	4710	4805	471	29	3,6%	3,6%	5,5%	3,6%
**TOTAL**	**1828**	**12798**	**14626**	**1466**	**95**	**14199**	**107**	**15867**	**80,2%**	**110,9%**	**1,4%**	**108,5%**	**16160**	**16170**	**16514**	**1616**	**129**	**1,8%**	**1,9%**	**3,9%**	1,8%

MVD: Measles Vaccine Diluent; SB and Inc. Number of safety boxes used and incinerated; MV: Measles Vaccine; m = Months; ADS: Auto-disabled Syringes;%: Percentage, NHD: Nylon Health District.

#### Specific vaccination around cases

These interventions were carried out around 25 measles cases notified from neighborhoods in the four health areas not involved in the immunization response campaign. A total of 824 children aged 9 to 59 months were vaccinated against measles during these outreach activities hence an average of 33 children per outreach with a range of 4 to 60. Most of the vaccinated children 656(79.6%) were aged 12 to 59 months.

#### Comparative epidemic curve

**Figure 1:**
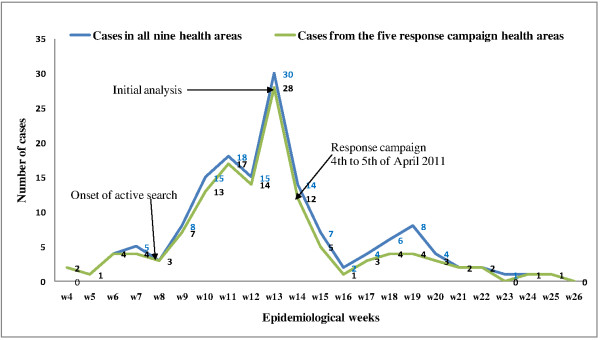
Epidemic curve for all 9 health areas and 5 health areas with measles outbreak immunization response NHD 2011.

The outbreak lasted for 21 epidemiological weeks (starting from the 4^th^ to the 25^th^ epidemiological week of 2011) as illustrated in Figure 1. The immunization response campaign took place on the 14^th^ epidemiological week, exactly 10 weeks after the onset of the outbreak which later ended 11 weeks after the onset of the response. A total of 153 cases reported by all nine health areas during the outbreak respected the WHO measles case definition amongst which 115(75.2%) were before the immunization response campaign. The five health areas that benefitted from the immunization response campaign reported 131 cases in all, with 105(80.2%) before the 14^th^ epidemiological week. After the response campaign, the gap between both curves widens compared to the closeness at the beginning of the outbreak. A total of 29 cases were reported by all nine health areas from 14 days post campaign amongst which 20(70%) were in the five health areas that benefitted from the immunization response campaign.

## Conclusion

Much can be done with existing resources available at regional and operational level during emergency situations like outbreaks. The timely intervention locally initiated and executed by the Littoral region contributed enormously in reducing the duration, morbidity and mortality of the measles outbreak in the Nylon Health District. This initiative should be considered the way forward because it permitted the authorities of the region to organize the only measles vaccination response campaign out of the more than forty outbreaks registered in the country in 2011. Decision makers at the intermediate and operational levels should officially be instructed to redeploy funds during emergency situations so as to prevent the development of extreme public health conditions by controlling them on time.

## Competing interest

The authors declare that they have no competing interests.

## Authors’ contribution

MK and IE identified the outbreak and informed the region. Together with GES and AABF they defined strategies and developed data collection tools. GES, SN and PA trained the personnel of the district on elaborated strategies and the use of data collection stools. AABF designed the proposal and coordinated the response campaign. SN and PA carried out active search of cases in the community. All the authors participated in supervising response activities and designing the study. Data analysis and the production of the first draft was done by GES. The article was completed and approved by all authors.
